# Prevention and treatment of surgical site infection in HIV-infected patients

**DOI:** 10.1186/1471-2334-12-115

**Published:** 2012-05-14

**Authors:** Lei Zhang, Bao-Chi Liu, Xiao-Yan Zhang, Lei Li, Xian-Jun Xia, Rui-Zhang Guo

**Affiliations:** 1 Surgery Department, Shanghai Public Health Clinical Center affiliated to Fudan University, Shanghai, 201508, China; 2 Scientific Research Center, Shanghai Public Health Clinical Center affiliated to Fudan University, Shanghai, 201508, China

## Abstract

**Background:**

Surgical site infection (SSI) are the third most frequently reported nosocomial infection, and the most common on surgical wards. HIV-infected patients may increase the possibility of developing SSI after surgery. There are few reported date on incidence and the preventive measures of SSI in HIV-infected patients. This study was to determine the incidence and the associated risk factors for SSI in HIV-infected patients. And we also explored the preventive measures.

**Methods:**

A retrospective study of SSI was conducted in 242 HIV-infected patients including 17 patients who combined with hemophilia from October 2008 to September 2011 in Shanghai Public Health Clinical Center. SSI were classified according to Centers for Disease Control and Prevention (CDC) criteria and identified by bedside surveillance and post-discharge follow-up. Data were analyzed using SPSS 16.0 statistical software (SPSS Inc., Chicago, IL).

**Results:**

The SSI incidence rate was 47.5% (115 of 242); 38.4% incisional SSIs, 5.4% deep incisional SSIs and 3.7% organ/space SSIs. The SSI incidence rate was 37.9% in HIV-infected patients undergoing abdominal operation. Patients undergoing abdominal surgery with lower preoperative CD4 counts were more likely to develop SSIs. The incidence increased from 2.6% in clean wounds to 100% in dirty wounds. In the HIV-infected patients combined with hemophilia, the mean preoperative albumin and postoperative hemoglobin were found significantly lower than those in no-SSIs group (P<0.05).

**Conclusions:**

SSI is frequent in HIV-infected patients. And suitable perioperative management may decrease the SSIs incidence rate of HIV-infected patients.

## Background

Surgical site infection (SSI) are the third most frequently reported nosocomial infection, and the most common on surgical wards [1]. The Centers for Disease and Prevention (CDC) estimates that 22% of all the health-care-associated infections are SSI, with an increasing percentage over the last decennium [1,2]. SSI increase morbidity as well as mortality, double the length of hospital stay [3-6] and increase the cost of surgery two-fivefold [7]. Kirkland et al. showed that 60% of the patients who develop an SSI are more likely to be admitted to an intensive care unit (ICU), or to be hospitalized for another 5 days and more than five times more likely to be re-admitted. Therefore, SSI rates are increasingly seen as a performance indicator for the quality of health care [8]. In 2009, the World Health Organization issued guidelines on how to prevent SSI in developed as well as in developing countries [9].

Human immunodeficiency virus (HIV)/acquired immune deficiency syndrome (AIDS) is a worldwide pandemic. In recent years, the number of HIV-infected patients is progressively increasing. The introduction of highly active antiretroviral therapy (HAART) has significantly improved the life expectancy of patients infected with HIV and those diagnosed with AIDS [10-15]. The demand for surgical treatment in HIV-infected patients combined with surgical disease is on the rise [16,17]. The progressive failure of the immune system in patients caused by HIV can increase the possibility of developing surgical site infections after surgery. The aim of this study was to determine the incidence and the associated risk factors for SSI in HIV-infected patients. And we also explored the preventive measures.

## Methods

### Definitions

Centers for Disease Control and Prevention (CDC) definition for wound class and classification of SSI were used. Surgical site infection (SSI) was defined as an infection occurring within 30 days of the operative procedure, with the patient having one or more of the followings: (i) purulent drainage from the surgical incision; (ii) organisms isolated from an aseptically obtained culture of fluid or tissue from the surgical incision; (iii) at least one of the following: pain or tenderness, localized swelling, redness, or heat. SSIs were classified as being incisional, deep incisional or organ/space. A surgical procedure was defined as the single surgery that was performed in the operating theatre. If more than two surgical procedures were performed in a single patient, the characteristics of the first surgical procedure were described.

### Data collection

Clinical data on HIV-infected patients undergoing surgery from October 2008 to September 2011 were retrieved using our computerized patient record system. Inclusion criteria: HIV-infected patients were identified and diagnosed by local Centers for the Disease Prevention and Control in different places. HIV test was done using ELISA and Western blot. Upon admission, all selected patients had records containing information on thorough disease histories, physical examinations, preoperative and postoperative routine examination, and immune function tests.

### Patient group and study methods

A retrospective study of SSI was conducted in 242 HIV-infected patients including 17 patients who combined with hemophilia from October 2008 to September 2011 in Shanghai Public Health Clinical Center. SSI were classified according to Centers for Disease Control and Prevention (CDC) criteria and identified by bedside surveillance and post-discharge follow-up. We stratified and compared the incidence of SSIs according to preoperative CD4 counts with breakpoint values of 200 and wound class. Patients combined with hemophilia were divided into SSIs group (A) and no-SSIs group (B). Demographic and clinical information was entered into a database that included: type of surgical procedure, age, peripheral blood cells, plasma albumin, CD4 counts, and CD4/CD8 ratios.

Our study was a retrospective study conformed to the tenets of the Declaration of Helsinki. It had been approved by Institutional Ethics Review Board of Shanghai Public Health Clinical Center (International index IORG0006364).

### Statistical analysis

Data were analyzed using SPSS 16.0 statistical software (SPSS Inc., Chicago, IL). Results of all continuous data are presented here as mean ± standard deviation. Continuous variables were compared with independent *t*-test. Univariate analysis of the categorical outcome was carried out using Chi-squared tests. P<0.05 indicates statistical significance.

## Results

Two hundred and forty-two HIV-infected patients were included in this study. Their mean (SD) age was 42 ± 13 years, and 220 (90.9%) were male. There were four deaths in the study population, all of whom had developed organ/space SSI and sever sepsis. The SSI cumulative incidence rate was 47.5% (115 of 242). The incidence of superficial incisional SSI was 38.4%, deep incisional SSI was 5.4%, and organ/space SSI was 3.7%. The types of surgical operations are listed in Table 1.

**Table 1:** The types of surgical operations and SSI in each procedures

**Surgical procedures**	**No-SSIs**	**Incisional** **SSI**	**Deep incisional SSI**	**Organ/space** **SSI**	**Total**
Perianal Surgery	4	55	0	0	59
Purulent Drainage	2	8	6	4	20
Orthopedic Surgery	20	6	1	0	27
Lymphadenectomies	12	3	0	0	15
Partial Hepatectomy	12	2	1	1	16
Colon Surgery	8	10	1	1	20
Skin Tumor Removal	20	2	0	0	22
Amputation	0	1	1	0	2
Splenectomy	7	0	0	1	8
Thyroidectomy	9	0	0	0	9
Appendectomy	2	2	0	0	4
Gastric Surgery	3	0	0	0	3
Tumor Resection	7	0	0	1	8
Hernia	4	0	0	0	4
Urinary Operation	7	1	0	1	9
Others	10	3	3	0	16
Total	127	93	13	9	242

Comparisons of the incidence of SSIs according to preoperative CD4 counts are shown in Table 2 and Table 3. Patients with lower preoperative CD4 counts undergoing abdominal surgery were more likely to develop SSIs. In total patients, no significant differences were seen according to preoperative CD4 counts.

**Table 2:** Incidence of SSIs in patients undergoing abdominal surgery stratified by preoperative CD4 counts

**Group**	**SSI(total = 25), N(%)**	**no-SSI(total = 41), N(%)**	**P**	**OR**	**95%CI**
<200	16(64%)	14(34.1%)		1	
≥200	9(36%)	27(65.9%)	0.02	0.292	0.103-0.826

**Table 3:** Comparisons of the Incidence of SSIs in all patients

**Group**	**SSI, N(%)**	**no-SSI, N(%)**	**P**	**OR**	**95%CI**
Preoperative CD4 Counts	Group				
<200	47(46.5%)	47(42.7%)		1	
≥200	54(53.5%)	63(57.3%)	0.087	0.796	0.739-0.857
Wound class					
Clean	2(1.7%)	75(59.1%)		1	
Contaminated	102(88.7%)	52(40.9%)	<0.001	71.87	16.96-304.48
Dirty	11(9.6%)	0	<0.001	76.71	18.24-322.68

Surgical procedures were classified as clean (N = 77; 31.8%), contaminated (N = 154; 63.6%) and dirty (N = 11; 4.5%). The incidence of SSI differed significantly depending on wound class, and increased from 2.6% in patients with clean wounds to 100% in patients with dirty wound(Table 3). Our data show that SSIs were frequent and differed widely by wound class.

Patients combined with hemophilia were divided into SSIs group (A) and no-SSIs group (B). Comparisons of clinical date between two groups are given in Table 4. In the SSIs group, the mean preoperative albumin and postoperative hemoglobin were found significantly lower than those in no-SSIs group (P<0.05). Other laboratory variables were not seen different between two groups (P>0.05).

**Table 4:** **Comparison of Clinical** Data **between Group A and Group B**

**Parameters**	**Group A**	**Group B**	**t**	**P**
Preoperative				
CD4 (cell/μ l)	382.1 ± 184.1	382.0 ± 111.3	−0.002	0.999
CD8 (cell/μ l)	609.4 ± 362.9	743.0 ± 318.9	0.717	0.487
CD4/CD8	0.7476 ± 0.361	0.6012 ± 0.295	−0.809	0.434
WBC(x109/L)	6.51 ± 2.79	4.72 ± 1.53	−1.521	0.151
Hemoglobin (g/L)	123.7 ± 25.5	142.7 ± 31.1	1.344	0.200
Platelet(x109/L)	176.5 ± 120.1	267 ± 131.7	1.435	0.173
Albumin (g/L)*	39.9 ± 5.2	46.7 ± 1.3	3.792	0.004
Postoperative				
CD4 (cell/μl)	382.6 ± 186.1	388.4 ± 91.4	0.063	0.952
CD8 (cell/μl)	498.6 ± 67.3	723 ± 358.4	1.376	0.206
CD4/CD8	0.766 ± 0.355	0.652 ± 0.329	−0.527	0.613
WBC(x109/L)	7.895 ± 4.668	5.84 ± 1.92	−1.143	0.281
Hemoglobin (g/L)*	103.4 ± 24.5	134.8 ± 27.2	2.354	0.035
Platelet(x109/L)	169.1 ± 48.5	226.3 ± 133.7	1.071	0.318
Albumin (g/L)	40.0 ± 5.5	43.9 ± 3.3	1.657	0.122

Group A = SSI; Group B = no-SSI; WBC = white blood cell.

*Statistical significance.

## Discussion

The progressive failure of the immune system in patients caused by HIV can increase the possibility of developing surgical site infections after surgery. Our study demonstrated an incidence of 47.5% SSIs in HIV-infected patients. This number is above the average SSI rate of 2.61% by the NNIS review [18]. Drapeau CM et al. showed the SSI rate of HIV-infected patients was twofold than that reported in Italian and European studies for general population [19]. In abdominal operations, patients’ mean preoperative CD4 counts in the SSIs group were found significantly lower than those in no-SSIs group, suggesting it may be associated with SSIs. Mawalla et al. also observed the rate of SSI among HIV patients was significantly higher with CD4 count below 200 cell/μ L[20]. It is universally accepted that the CD4 counts is a valuable marker of disease progression in HIV and AIDS. Generally, when CD4 counts >350cells/μ L, there is no obvious damage to the immune function, patients’ treatment and range of indications for surgery can be same as normal surgical patients without HIV infected; when CD4 counts is between 200–350 cells/μ L, patients need to examine body completely to find whether merge other complications and start highly active anti-retrovirus therapy (HAART). And for those patients whose preoperative CD4 counts ≤ 200 cells/μ L, the antibiotic and antifungal medications (sulfamethoxazole/trimethoprim (SMZ/TMP) and fluconazole) are started preoperatively as prophylaxis against Pneumocystis carinii pneumonia (PCP) and fungal infection.

In our opinion, the evaluation of the risk of developing SSIs for HIV-infected patients should be an integral part of the perioperative management. Although the optimum time for surgery still needs clarification in further studies, it may be better to delay elective surgery until the preoperative CD4 count>200cells/μ L. However, in patients with typically surgical problems, e.g., appendicitis and gastrointestinal perforation, early surgery allows for rapid recovery similar to normal surgical patients irrespective of CD4 count.

Our data show that SSIs were frequent and differed widely by wound class. Wound care efore operations for contaminated and dirty wounds should be done with more caution. Further strengthening of basic infection control in the hospital to improve the hospital environment is also required. Another important intervention will be required to encourage surgeons to use appropriate antibiotic prophylaxis. Petrosillo N et al. found that antibiotic prophylaxis was also associated with postdischarge SSI [21].

In the HIV-infected patients combined with hemophilia, there was a clinically significant decrease of postoperative hemoglobin count in the SSIs group compared with the no-SSIs group, which is mainly associated with SSIs. Bleeding is the major risk of intraoperation and postoperation for hemophilia . Infusing 2000 units of factor VII 2 times one day before surgery, 2000 units of factor VII 2 hours before operation, 2000–4000 units during operation, can improve patients’ coagulation to normal levels.

One of the limitations of the study was information biases due to the retrospective nature of the design study. The other was that it did not control for possible confounders other than those investigated.

## Conclusions

Postoperative SSIs are frequent in HIV-infected patients. It appears necessary to establish a reliable and efficient national monitoring programme for the implementation of preventive measures.

## Competing interests

The authors declare that they have no competing interests.

## Authors’ contributions

LZ, BCL and XYZ conceived of the study, designed and drafted the manuscript. LL, XJX and GRZ participated in data collection. All authors read and approved the final manuscript.

## Pre-publication history

The pre-publication history for this paper can be accessed here:

http://www.biomedcentral.com/1471-2334/12/115/prepub
